# Comprehensive nursing interventions enhance sleep quality in patients with arrhythmia following AMI

**DOI:** 10.1097/MD.0000000000041182

**Published:** 2025-01-17

**Authors:** Li Song, Yan Xiong, Hanxiang Gao, Suyu Yao, Hua Deng

**Affiliations:** a Department of Cardiology, The First Hospital of Lanzhou University, Lanzhou, China; b Department of Respiratory Medicine (Geriatrics), Wuhan Wuchang Hospital, Wuhan, China; c Quality Management Office, Wuhan Wuchang Hospital, Wuhan, China.

**Keywords:** arrhythmia after acute myocardial infarction, comprehensive nursing, rehabilitation time, sleep quality

## Abstract

This study aims to assess the impact of comprehensive nursing care on sleep quality and rehabilitation duration in patients experiencing arrhythmia after acute myocardial infarction (AMI). Eighty-four patients with post-AMI arrhythmia treated at our hospital from February 2018 to February 2019 were selected and divided based on the nursing care received. The observation group (n = 44) underwent comprehensive nursing interventions, while the control group (n = 40) received standard nursing care. Evaluations of cardiac function indices and sleep quality were conducted before and after the nursing interventions. In addition, comparisons were made between the 2 groups regarding arrhythmia occurrence, patient compliance during care, visual analog scale scores for pain, self-rating anxiety scale scores, self-rating depression scale scores, and overall nursing satisfaction. Following the nursing interventions, the observation group exhibited a significant increase in cardiac output, unlike the control group, which showed no notable change. Both groups demonstrated significant improvements in left ventricular end-diastolic dimension and left ventricular ejection fraction; however, these enhancements were more pronounced in the observation group. Post-intervention, the Pittsburgh Sleep Quality Index scores were significantly lower in the observation group compared with the control group, indicating better sleep quality. The observation group also experienced a significant reduction in the incidence, frequency, and duration of arrhythmia episodes. Furthermore, this group showed a lower incidence of complications during the intervention period and reported lower visual analog scale, self-rating anxiety scale, and self-rating depression scale scores after nursing care. Nursing satisfaction rates were notably higher in the observation group than in the control group. Comprehensive nursing care significantly enhances sleep quality, speeds up rehabilitation, and increases patient satisfaction in individuals with arrhythmia after AMI. These findings support the clinical adoption of comprehensive nursing interventions for this patient population.

## 1. Introduction

Patients with acute myocardial infarction (AMI) face risks of life-threatening ventricular arrhythmia (VA), cardiac arrest, hemodynamic instability, and cardiogenic shock (CS).^[1]^ Arrhythmia is a common symptom of acute coronary syndrome and a common severe complication of AMI,^[2]^ and it increases the risk of death.^[3]^ Ventricular arrhythmia and sudden cardiac death caused by AMI are common causes of death in human beings. Reportedly, over 10% of cases with AMI have suffered from fatal VA such as ventricular fibrillation before hospitalization, and their survival rate is extremely low.^[4]^ Therefore, early treatment and clinical nursing are of great significance to patients’ rehabilitation.

As economy develops, people enjoy constantly improved life and demand higher levels of medical care and services, so the traditional nursing model centered on disease is unable to meet the nursing needs of patients gradually.^[5]^ Comprehensive nursing mode is a novel nursing mode with people as the core, under which factors that affect the prognosis of patients, such as physical fitness, psychological state, and disease development, are comprehensively evaluated, and then an organized and planned personalized nursing plan is developed for the patients to improve their prognosis and life quality.^[6,7]^ At this point, there are many researches doing well in studying the clinical application of comprehensive nursing. For example, 1 study has revealed that for patients with diabetes mellitus, comprehensive nursing can effectively improve the nursing against diabetes mellitus, promote patients’ self-management, and reduce the risk of cardiovascular disease.^[8]^ In 1 other study, the effects of comprehensive nursing and routine nursing on acute perioperative delirium in elderly patients with hip fracture were compared, and the results show that comprehensive nursing can notably reduce the incidence of perioperative delirium.^[9]^ In addition, there are also research results that comprehensive nursing intervention can improve the clinical efficacy on and life quality of patients with breast cancer.^[10]^

However, there is limited research on the application of comprehensive nursing in patients with arrhythmia after AMI. This study hypothesizes that comprehensive nursing can significantly improve the clinical outcomes and life quality of patients with arrhythmia after AMI compared with routine nursing. To test this hypothesis, the study explored the actual value of comprehensive nursing by comparing it with routine nursing, aiming to provide a better nursing plan for disease management and recovery.

## 2. Clinical materials and methods

### 2.1. Collection of clinical data

The study was approved by the Ethics Committee of the First Hospital of Lanzhou University. A total of 84 patients with arrhythmia after AMI treated in our hospital from February 2018 to February 2019 were enrolled and grouped according to different nursing methods. Among them, 44 patients were intervened by comprehensive nursing as an observation group, while other 40 patients were intervened by routine nursing as a control group. This study was approved by the Medical Ethics Committee of our hospital.

### 2.2. Inclusion and exclusion criteria of patients

#### 2.2.1. The inclusion criteria

Patient diagnosed as arrhythmia after AMI according to electrocardiogram (ECG) based on related ACC/AHA/HRS guidelines released in 2015,^[11]^ patients with detailed clinical data, patients with education level above primary school, and patients who and whose family members signed informed consent forms.

#### 2.2.2. The exclusion criteria

Endangered patients, patients with other comorbid heart diseases, patients with malignant tumors, communication obstacle, or mental disorder, and those during pregnancy or lactation.

### 2.3. Nursing methods

Patients in the control group received standard nursing care commonly administered to individuals with arrhythmia following AMI. This included medication guidance, where nurses informed patients of prescribed drug regimens, emphasizing adherence to dosage and timing instructions. Dietary guidance was also provided, focusing on general recommendations such as reducing salt and fat intake to support cardiovascular health. In addition, monitoring of vital signs was conducted routinely to track parameters such as heart rate, blood pressure, and oxygen saturation. Nurses maintained a consistent schedule of monitoring to detect any significant changes in the patients’ condition. However, routine care did not involve personalized psychological support, environmental modifications, or structured rehabilitation plans, making it less comprehensive compared with the intervention group.

Patients in the observation group were given comprehensive nursing by nursing staff in our hospital who had accepted multidisciplinary learning on the basis of routine nursing. The specific operations were as follows: health education: nursing staff provided systematic education to patients and their families using tailored materials such as brochures, videos, and educational sessions that explained the causes, progression, and management of arrhythmia post-AMI. Interactive methods like Q&A sessions, role-playing (e.g., demonstrating safe limb movements), and hands-on workshops were used to enhance understanding and engagement. Education was delivered daily during hospitalization, with a review session before discharge to ensure comprehension and promote treatment adherence; psychological support: nurses conducted initial psychological assessments using standardized tools such as the self-rating anxiety scale (SAS) and self-rating depression scale (SDS) to identify patients’ emotional states. Based on these evaluations, personalized psychological counseling plans were developed and implemented through daily 15- to 20-minute sessions. These sessions focused on relaxation techniques, stress management, and motivational reinforcement, encouraging patients to adopt a positive mindset and actively participate in their recovery process; environmental optimization: to create a calming hospital environment, patients were placed in single or semiprivate rooms with noise-reduction features and dimmable lighting. Lavender aromatherapy was used to promote relaxation, and soothing instrumental music was played for 30 to 60 minutes daily, especially during rest periods. These measures aimed to reduce environmental stressors, improve mood, and enhance sleep quality; advanced medication guidance: patients and their families received step-by-step guidance on medication use, including timing, dosage, potential side effects, and management of adverse reactions, supported by visual aids like illustrated charts and mobile apps. Weekly adherence checks were performed through interviews and pill counts to ensure proper medication use. This process aimed to reduce the risk of complications and improve long-term health outcomes; pain management: nurses employed nonpharmacological interventions, such as adjusting body positions to alleviate chest pain and guiding patients through relaxation techniques like controlled breathing exercises. Heat packs were used for muscle tension relief, and transcutaneous electrical nerve stimulation was applied when needed. For severe pain, prescribed analgesics were administered promptly, ensuring both comfort and the ability to engage in rehabilitation activities; complication prevention: vital signs and ECGs were continuously monitored during the critical 72-hour post-AMI period, with immediate action plans for any abnormal events. Bedside reminders, including visual signs and verbal instructions, emphasized avoiding physical exertion, particularly during defecation. These proactive measures minimized the occurrence of complications and improved patient safety; nutritional support: patients were provided with personalized meal plans developed by dietitians, tailored to their comorbidities and dietary preferences. The plans focused on high-protein, low-fat, and easily digestible foods while avoiding substances like caffeine and high-sodium items that could exacerbate arrhythmia. Nursing staff offered additional guidance on food preparation and portion control, involving family members when possible to ensure continuity after discharge; rehabilitation training: rehabilitation activities were introduced in stages, starting with passive range-of-motion exercises assisted by nursing staff and progressing to light, self-initiated exercises such as limb stretching and supervised ambulation. Sessions were conducted twice daily for 10 to 20 minutes, with intensity gradually increasing as tolerated. These activities promoted blood circulation, reduced the risk of thrombosis, and strengthened physical recovery.

### 2.4. Outcome measures

#### 2.4.1. Main outcome measures

The levels of heart function indexes including cardiac output (CO), left ventricular end-diastolic dimension (LVEDD), and left ventricular ejection fraction (LVEF) of the 2 groups before and after nursing were evaluated, and the occurrence of arrhythmia during nursing in the 2 groups were analyzed. In addition, the bed rest time and length of stay of the 2 groups were counted. The Pittsburgh Sleep Quality Index (PSQI) was adopted to evaluate and compare the sleep quality of patients in the 2 groups.^[12]^ A higher PSQI score indicates worse sleep quality.

#### 2.4.2. Secondary outcome measures

The occurrence of complications in the 2 groups during nursing was analyzed, and the visual analog scale (VAS), SAS, and SDS scores of the 2 groups were evaluated. In addition, the self-made Nursing Satisfaction Questionnaire of our hospital was used to evaluate nursing satisfaction of the patients, which mainly covered attitude, character, wearing, and operation proficiency. The questionnaire consisted of 20 questions, and each question was worth 5 points. The score <70 points indicated dissatisfaction, score between 70 and 89 indicated basic satisfaction, and score ≥90 points indicated satisfaction. Satisfaction = (the number of patients satisfied with nursing + the number of patients moderately satisfied with nursing)/the total number of patients × 100%.

### 2.5. Statistical analyses

In this study, the medical statistics analysis software SPSS20.0 (Chicago SPSS Company) was adopted to statistically analyze the collected data, and GraphPad Prism 7 (San Diego Graphpad Software Co, Ltd) was used to visualize the data into required figures. The utilization rate of enumeration data (%) was analyzed using the χ^2^ test, and expressed by ×2. Measurement data were expressed as the mean ± standard deviation (mean ± SD). All measurement data were in normal distribution. Comparison between 2 groups was performed using the independent-samples *t* test, and comparison within groups was carried out using the paired *t* test. *P* < .05 indicates a significant difference.

## 3. Results

### 3.1. Comparison of general clinical data

According to comparison of the 2 groups in general clinical data, no notable difference was found between them in age, sex, body mass index, myocardial infarction site, arrhythmia frequency, arrhythmia duration, education level, smoking history, history of heavy drinking, and place of residence (all *P* > .05; Table 1).

**Table 1:** Comparison of general clinical data.

Factor	The observation group (n = 44)	The control group (n = 40)	*t*/χ^2^	*P* value
Age	54.4 ± 8.3	55.8 ± 7.8	0.696	.489
Sex				
Male	26 (59.09)	22 (55.00)	0.808	.369
Female	18 (40.91)	18 (45.00)		
BMI (kg/m^2^)	20.81 ± 1.87	21.64 ± 1.75	0.430	.668
Myocardial infarction site			1.191	.275
Anterior wall	11 (25.00)	9 (22.50)		
Posterior wall	9 (20.45)	11 (27.50)		
Inferior wall	13 (29.55)	8 (20.00)	0.778	.378
Lateral wall	7 (15.91)	7 (17.50)		
Others	4 (9.09)	5 (12.50)		
Arrhythmia frequency (d)	3658.6 ± 125.5	3678.4 ± 138.2	0.688	.493
Arrhythmia duration (min)	7.55 ± 0.42	7.61 ± 0.45	0.632	.529
Education level				
With junior high school diploma or below	19 (43.18)	20 (50.00)	0.474	.491
With senior high school diploma and above	25 (56.82)	20 (50.00)		
Smoking history				
Yes	31 (70.45)	32 (80.00)	1.120	.290
No	13 (29.55)	8 (20.00)		
History of heavy drinking				
Yes	28 (63.64)	23 (57.50)	0.309	.578
No	16 (36.36)	17 (42.50)		
Place of residence				
Urban area	24 (54.55)	18 (45.00)	0.205	.651
Rural area	20 (45.45)	22 (55.00)		

*P < .05 was considered significant, and *P* was corrected by Bonferroni.

### 3.2. Comparison of heart function indexes

**Figure 1.**
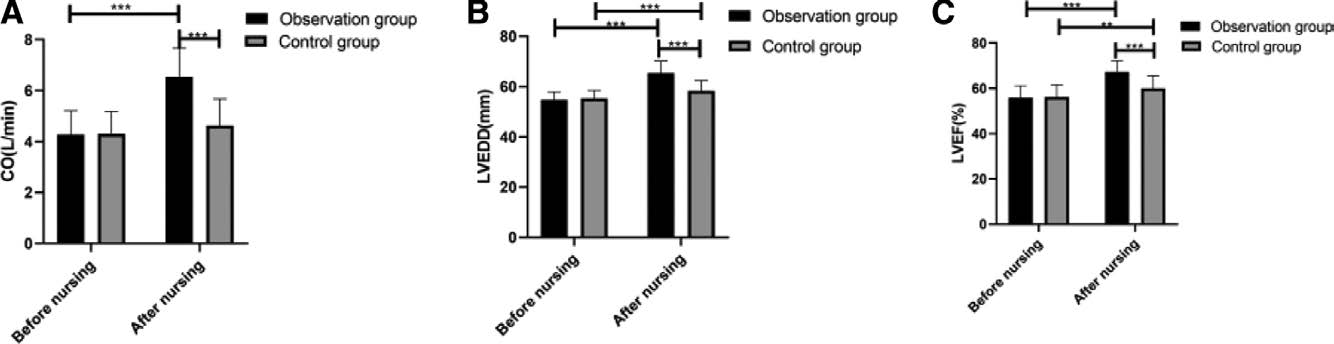
Comparison of heart function indexes. (A) Before nursing, there was no notable difference in CO level between the 2 groups, while after nursing, the CO level of both groups increased significantly, and the CO level of the observation group was notably higher than that of the control group. (B) Before nursing, there was no notable difference in LVEDD between the 2 groups, while after nursing, LVEDD of both groups increased significantly, and LVEDD of the observation group was notably higher than that of the control group. (C) Before nursing, there was no notable difference in LVEF between the 2 groups, while after nursing, LVEF of both groups increased significantly, and LVEF of the observation group was notably higher than that of the control group. **P < .01 and ***P < .001. CO = cardiac output, LVEDD = left ventricular end-diastolic dimension, LVEF = left ventricular ejection fraction.

According to the determination of heart function indexes in the 2 groups, before nursing, no notable difference was found between the observation group and the control group in CO, LVEDD, and LVEF, while after nursing, the CO of the observation group increased significantly, that of the control group did not change notably, LVEDD and LVEF of both groups increased notably, and LVEDD and LVEF of the observation group were both notably higher than those of the control group (Fig. 1).

### 3.3. Occurrence of arrhythmia

According to comparison between the 2 groups in the occurrence of arrhythmia, after nursing, the incidence of arrhythmia in the observation group was notably lower than that in the control group, and the frequency and duration of arrhythmia in the observation group were also notably lower than those in the control group (Table 2).

**Table 2:** Comparison of arrhythmia occurrence between the 2 groups.

Group	Cases of arrhythmia	Arrhythmia frequency	Arrhythmia duration
The observation group (n = 44)	6 (13.64)	916.3 ± 114.5	3.12 ± 0.24
The control group (n = 40)	13 (32.50)	1207.4 ± 130.6	4.91 ± 0.91
*t*/χ^2^	4.260	10.88	12.58
*P* value	.039	<.001	<.001

*P < .05 was considered significant, and *P* was corrected by Bonferroni.

### 3.4. Occurrence of complications

There were 2 cases of fever, 1 case of heart failure, and 1 case of myocardial infarction in the observation group, while there were 3 cases of fever, 4 cases of heart failure, 2 cases of myocardial infarction, and 2 cases of CS in the control group, so the total incidence of complications in the observation group was notably lower than that in the control group (Table 3).

**Table 3:** Comparison between the 2 groups in the occurrence of complications.

Group	Fever	Heart failure	Myocardial infarction	Cardiogenic shock	The total incidence
The observation group (n = 44)	2 (4.55)	1 (2.27)	1 (2.27)	0 (0.00)	4 (9.09)
The control group (n = 40)	3 (7.50)	4 (10.00)	2 (5.00)	2 (5.00)	11 (27.50)
χ^2^					4.841
*P* value					.028

*P < .05 was considered significant, and *P* was corrected by Bonferroni.

### 3.5. Comparison of sleep quality

According to comparison of PSQI scores between the 2 groups, before nursing, no notable difference was found between the 2 groups in PSQI score, while after nursing, the PSQI score of the observation group was notably lower than that of the control group (Table 4).

**Table 4:** Comparison of PSQI score between the 2 groups.

	PSQI score	
Group	Before nursing	After nursing
The observation group (n = 44)	13.84 ± 2.15	6.12 ± 1.13
The control group (n = 40)	14.10 ± 2.21	9.76 ± 1.25
*t*	.546	13.95
*P* value	.586	<.001

PSQI = Pittsburgh Sleep Quality Index.

*P < .05 was considered significant, and *P* was corrected by Bonferroni.

### 3.6. Comparison of rehabilitation time

**Figure 2.**
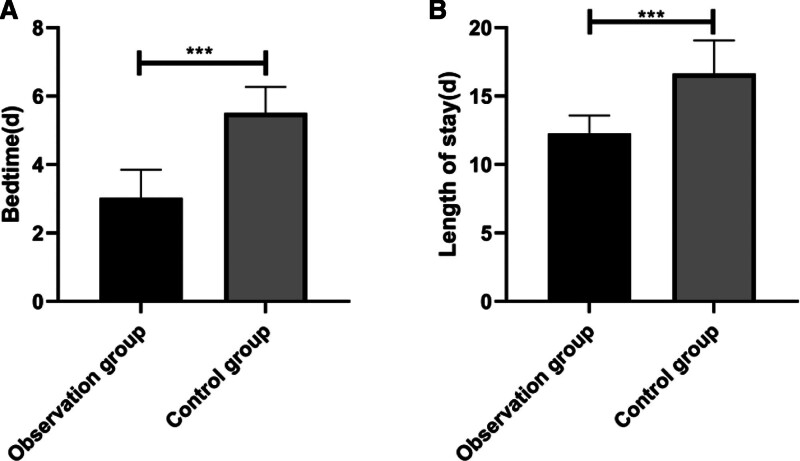
Comparison of bed rest time and length of stay between the 2 groups. (A) The observation group experienced notably shorter bed rest than the control group. (B) The observation group experienced notably shorter length of stay than the control group. * *P* < .05, ** *P* < .01, and *** *P* < .001.

According to analysis on the rehabilitation time of patients in the 2 groups, the observation group experienced notably shorter bed rest and length of stay than the control group (Fig. 2).

### 3.7. Comparison of VAS, SAS, and SDS scores

**Figure 3.**
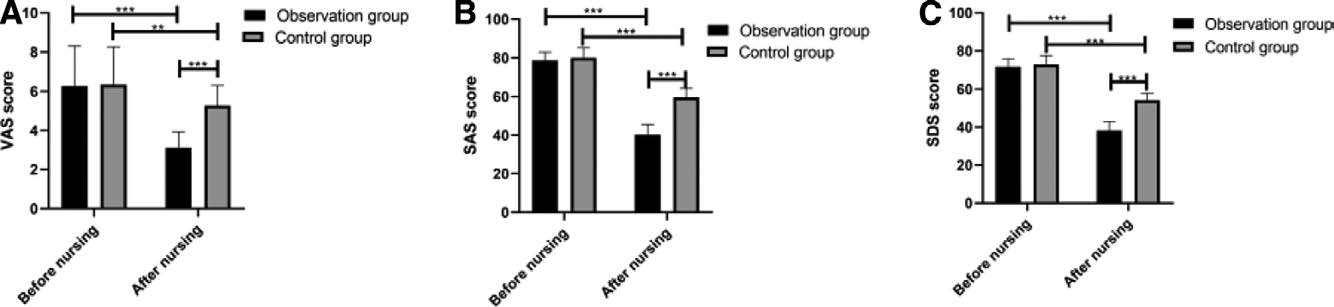
Comparison of VAS, SAS, and SDS scores between the 2 groups. (A) Before nursing, there was no significant difference in VAS score between the 2 groups, while after nursing, VAS scores of both groups decreased notably, and the VAS score of the observation group was notably lower than that of the control group. (B) Before nursing, there was no significant difference in SAS score between the 2 groups, while after nursing, SAS scores of both groups decreased notably, and the SAS score of the observation group was notably lower than that of the control group. (C) Before nursing, there was no significant difference in SDS score between the 2 groups, while after nursing, SDS scores of both groups decreased notably, and the SDS score of the observation group was notably lower than that of the control group. ** *P* < .01 and ***P < .001. VAS = visual analog scale, SAS = self-rating anxiety scale, SDS = self-rating depression scale.

According to comparison of pain situation and negative emotion between the 2 groups, before nursing, the VAS, SAS, and SDS scores of the observation group were not notably different from those of the control group, while after nursing, the VAS, SAS, and SDS scores of the 2 groups decreased notably, and the scores of the observation group were significantly lower than those of the control group (Fig. 3).

### 3.8. Comparison of nursing satisfaction

After nursing intervention, the observation group showed a nursing satisfaction of 88.64%, with 17 patients satisfied with nursing (38.64%), 22 patients moderately satisfied with nursing (50.00%), and 5 patients dissatisfied with nursing (11.36%), while the control group showed a nursing satisfaction of 67.50%, with 9 patients satisfied with nursing (22.50%), 18 patients moderately satisfied with nursing (45.00%), and 13 patients dissatisfied with nursing (32.50%), so the nursing satisfaction of the observation group was significantly higher than that of the control group (*P* < .05; Table 5).

**Table 5:** Comparison of nursing satisfaction between the 2 groups.

Group	Satisfaction	Moderate satisfaction	Dissatisfaction	Overall satisfaction
The observation group (n = 44)	17 (38.64)	22 (50.00)	5 (11.36)	39 (88.64)
The control group (n = 40)	9 (22.50)	18 (45.00)	13 (32.50)	27 (67.50)
χ^2^	-	-	-	5.560
*P* value	-	-	-	.018

*P < .05 was considered significant, and *P* was corrected by Bonferroni.

## 4. Discussion

Atrial fibrillation is the most common arrhythmia type in the United States and European countries. In Australia, European countries, and the United States, its prevalence rate is estimated to be 1% to 4%, while in Asia, it is estimated to be 0.49% to 1.9%. The prevalence rate of atrial fibrillation is in steady increase worldwide.^[13]^ In China, the total incidence of atrial fibrillation in patients with AMI during hospitalization is 3.0%.^[14]^ AMI complicated with arrhythmia usually aggravates myocardial ischemic injury and cardiac function injury and increases the risk of death.^[15]^ During AMI, atrial fibrillation occurs frequently, and the incidence of asymptomatic atrial fibrillation (16%) is 3 times that of symptomatic atrial fibrillation.^[16,17]^ The results of 1 long-term follow-up study show that the incidence of atrial fibrillation in patients with a AMI history is higher than that in the general population, and patients with a AMI history show a high cumulative incidence of it in 5 years after AMI (about 6%–21%), and face poor prognosis.^[18]^ Therefore, it is of great significance to carry out high-quality clinical nursing for patients with arrhythmia after AMI. Currently, there are few studies on the application of comprehensive nursing in patients with arrhythmia after AMI, and the influence of this nursing mode on nursing and rehabilitation effect of patients is still under investigation, so this study made explorations from these aspects to provide reference for clinical practice.

Statistics show that about 15% to 20% of patients with AMI have LVEF ≤ 35% during revascularization.^[19]^ LVEF and VA are considered as risk factors for identifying high-risk patients with arrhythmia after AMI, and the incidence of VA is inversely proportional to that of LVEF.^[20]^ We tested the heart function indexes of the 2 groups. It was turned out that after nursing, CO, LVEDD, and LVEF of the observation group were notably higher than those of the control group, indicating that comprehensive nursing had a more significant effect on improving cardiac function of patients with arrhythmia after AMI than routine nursing. We also counted the incidence of arrhythmia in the 2 groups, and found that the incidence of arrhythmia and frequency and duration of arrhythmia within 24 hours in the observation group were notably lower than those in the control group, implying that comprehensive nursing can reduce the incidence and frequency of arrhythmia after AMI and plays an essential role in patients’ rehabilitation. One study has revealed that the occurrence of VA after myocardial infarction is in accord with certain time law, and in animal models, it develops rapidly within the period of 90 minutes to 72 hours after infarction.^[21]^ Therefore, in this study, the vital signs and ECG of patients nursed under comprehensive nursing mode were strictly monitored during the 72-hour high incidence period after AMI, and patients were admonished or intervened when they have actions that might bring about arrhythmia. It was found that comprehensive nursing could effectively reduce the incidence of arrhythmia. CS is still the main cause of death of patients with AMI, with an incidence rate of 5% to 8%. Although a great progress has been achieved in treatment, the mortality rate of patients with AMI complicated with CS is still terribly high,^[22]^ so it is of great significance to prevent and monitor the complications of patients with arrhythmia after AMI. Therefore, we made statistics on the complications in the 2 groups during nursing, and found that the incidence of complications in the observation group was significantly lower than that in the control group. The results suggested that comprehensive nursing had a significant effect on reducing the incidence of complications in patients with arrhythmia after AMI. Patients who suffer from long-term chest pain or arrhythmia or receive partial revascularization are at a high risk of suffering from CS, and more than one third of patients with myocardial infarction and most patients with myocardial infarction complicated with CS face a higher risk of VA.^[23]^ In this study, comprehensive nursing demonstrated a good effect in preventing arrhythmia and CS, and promoted the improvement of patients’ condition.

Patients with arrhythmia usually have poor sleep quality, and usually suffer from insomnia and frequent nighttime awakening.^[24]^ Some patients with arrhythmia even have worse sleep quality due to negative emotion.^[25]^ Therefore, we evaluated the sleep quality of the 2 groups before and after nursing. It was turned out that before nursing, the PSQI scores of the 2 groups were not significantly different, and the scores were relatively high, indicating that patients with arrhythmia after AMI suffer from poor sleep before nursing. However, after nursing, the PSQI scores of both groups decreased, and the PSQI score of the observation group was notably lower than that of the control group. The results implied that comprehensive nursing was better than routine nursing in improving the sleep quality of patients. One study has pointed out that comprehensive nursing can effectively improve the sleep quality and life quality of women with sleep disorders, and has a positive effect on the health of patients.^[26]^ One study by Wei et al^[27]^ on the application of comprehensive nursing in patients undergoing minimally invasive percutaneous nephrolithotomy has also demonstrated the effectiveness of comprehensive nursing. It can significantly shorten operation and improve the success rate of operation and the sleep quality of patients. Although the research objects in this study are different from those in our study, the results also show that comprehensive nursing has a positive effect on sleep quality. Chronic pain compromises patients’ treatment compliance and quality of life.^[28]^ Patients with AMI are usually accompanied by chest pain.^[29]^ We evaluated the VAS scores of the 2 groups before and after nursing, finding that the VAS scores of the 2 groups were not notably different before nursing, while after nursing, the VAS scores of both groups decreased, and the VAS score of the observation group was notably lower than that of the control group, which showed that comprehensive nursing can effectively relieve patients’ pain. Patients usually suffer from negative emotion. Clinical research on patients with implantable defibrillators shows that anger will trigger VA, and long-term negative emotions will increase the risk of arrhythmia.^[30]^ Therefore, we evaluated the negative emotion of the 2 groups, and found that there was no notable difference in SAS and SDS scores between the 2 groups before nursing. The results indicated that the negative emotions of the 2 groups were serious before nursing, which may be caused by pain and poor sleep quality. After nursing, the SAS and SDS scores of both groups decreased notably, and scores of the observation group were notably lower than those of the control group, suggesting that comprehensive nursing can effectively ameliorate the negative emotions of patients and improve their mental health. One study by Lin and Zhang^[31]^ has showed that compared with routine nursing, comprehensive nursing can significantly ameliorate the anxiety and depression of patients with acute stroke during MRI examination, and has good application value in improving the examination completion rate, shortening examination, and improving nursing satisfaction, which is similar to the results of our study. We compared the bed rest time and length of stay between the 2 groups, finding that the observation group experienced shorter bed rest and length of stay than the control group, which implied that comprehensive nursing could greatly promote the rehabilitation of patients with AMI. One study by Zhang et al^[32]^ has shown that patients with stroke nursed under comprehensive nursing mode have better daily living ability, quality of life and compliance than those nursed under routine nursing mode. In our study, for patients nursed under comprehensive nursing, the nursing staff were arranged to encourage and help them to carry out rehabilitation training during treatment and eat under consideration of nutrition balance. We speculated that such measures effectively improved the self-management ability and treatment compliance of patients, and thus accelerated their rehabilitation. One study by Vaughan et al^[33]^ on the effect of comprehensive nursing on patients with Parkinson disease has revealed that comprehensive nursing is superior to routine nursing. One other study by Garvey et al^[34]^ has also shown that comprehensive nursing improves the overall health and quality of life of obese patients. At the end of this study, the nursing satisfaction of patients towards the 2 nursing modes was evaluated. It came out that comprehensive nursing received higher satisfaction than routine nursing, which also indicated that patients needed high-quality nursing and comprehensive nursing was worthy of clinical application.

This study has confirmed that comprehensive nursing can provide more benefits to patients with arrhythmia after AMI than routine nursing; however, it still has several limitations that should be addressed in future research. For instance, the study primarily focuses on short-term outcomes during hospitalization, such as improvements in cardiac function, sleep quality, and patient satisfaction, but lacks data on long-term outcomes, such as the recurrence rate of arrhythmia, rehospitalization rates, and sustained quality of life improvements. In addition, the study did not evaluate the patients’ adherence to lifestyle modifications and medication regimens after discharge, which are critical factors in managing chronic conditions like arrhythmia.

Moreover, the study’s sample size was relatively small and limited to a single center, which might reduce the generalizability of the findings to broader populations or different healthcare settings. Baseline characteristics between the groups were carefully compared and found to be well-balanced, which helped to partially control potential confounders. However, the use of univariate analysis may still limit the ability to fully account for residual confounding factors or interactions between variables. Future research should incorporate multivariate analyses, such as logistic or multiple linear regression, to better adjust for potential confounders and provide more robust evidence of the independent effects of comprehensive nursing interventions.

Furthermore, although psychological and environmental interventions were included in the comprehensive nursing protocol, specific mechanisms through which these interventions impacted outcomes were not explored in detail. Investigating these mechanisms in future studies could help refine and optimize nursing strategies. Lastly, exploring digital health tools, such as telemonitoring and mobile applications, could enhance post-discharge care, improve adherence, and provide valuable data on long-term outcomes.

## 5. Future directions

To overcome these limitations, future research should focus on conducting multicenter randomized controlled trials with larger sample sizes to confirm the findings and enhance generalizability. Long-term follow-up studies are also necessary to assess sustained benefits of comprehensive nursing, such as long-term cardiac function, quality of life, and the prevention of arrhythmia recurrence. Incorporating patient-reported outcomes, such as satisfaction with care and perceived health improvements, could provide a more holistic evaluation of the interventions.

In addition, future studies should explore the specific contributions of individual components of comprehensive nursing, such as psychological support or environmental modifications, to better understand their roles in improving patient outcomes. Finally, incorporating digital tools, such as mobile health applications or wearable devices, could enhance patient monitoring and support adherence to treatment plans, offering a more integrated and sustainable approach to post-discharge care.

## 6. Conclusion

To sum up, comprehensive nursing not only improves the sleep quality of patients with arrhythmia after AMI but also enhances cardiac function, accelerates recovery, and increases patient satisfaction with care. These findings highlight its potential as an effective and practical approach in clinical settings. Incorporating comprehensive nursing into routine care for patients with arrhythmia post-AMI could significantly improve short-term outcomes and potentially contribute to better long-term management. Future studies should focus on further validating these benefits and optimizing its implementation in diverse healthcare environments.

## Author contributions

**Conceptualization:** Li Song, Yan Xiong, Hanxiang Gao, Suyu Yao, Hua Deng.

**Data curation:** Li Song, Hanxiang Gao, Suyu Yao, Hua Deng.

**Formal analysis:** Li Song, Hanxiang Gao, Suyu Yao, Hua Deng.

**Investigation:** Li Song, Yan Xiong, Suyu Yao, Hua Deng.

**Methodology:** Li Song, Yan Xiong, Suyu Yao, Hua Deng.

**Validation:** Li Song, Yan Xiong.

**Writing—original draft:** Li Song, Hanxiang Gao.

**Writing—review & editing:** Li Song, Hanxiang Gao.

**Supervision:** Yan Xiong.

**Software:** Suyu Yao.
